# Supplementary material for: Compensatory mechanisms in γδ T cell-deficient chickens following Salmonella infection
Source: Front Immunol. 2025 May 14;16:1576766. doi: 10.3389/fimmu.2025.1576766 (PMC12117349; doi:10.3389/fimmu.2025.1576766)
Supplement: Supplementary file 1 [file DataSheet1.docx]

Supplementary Material


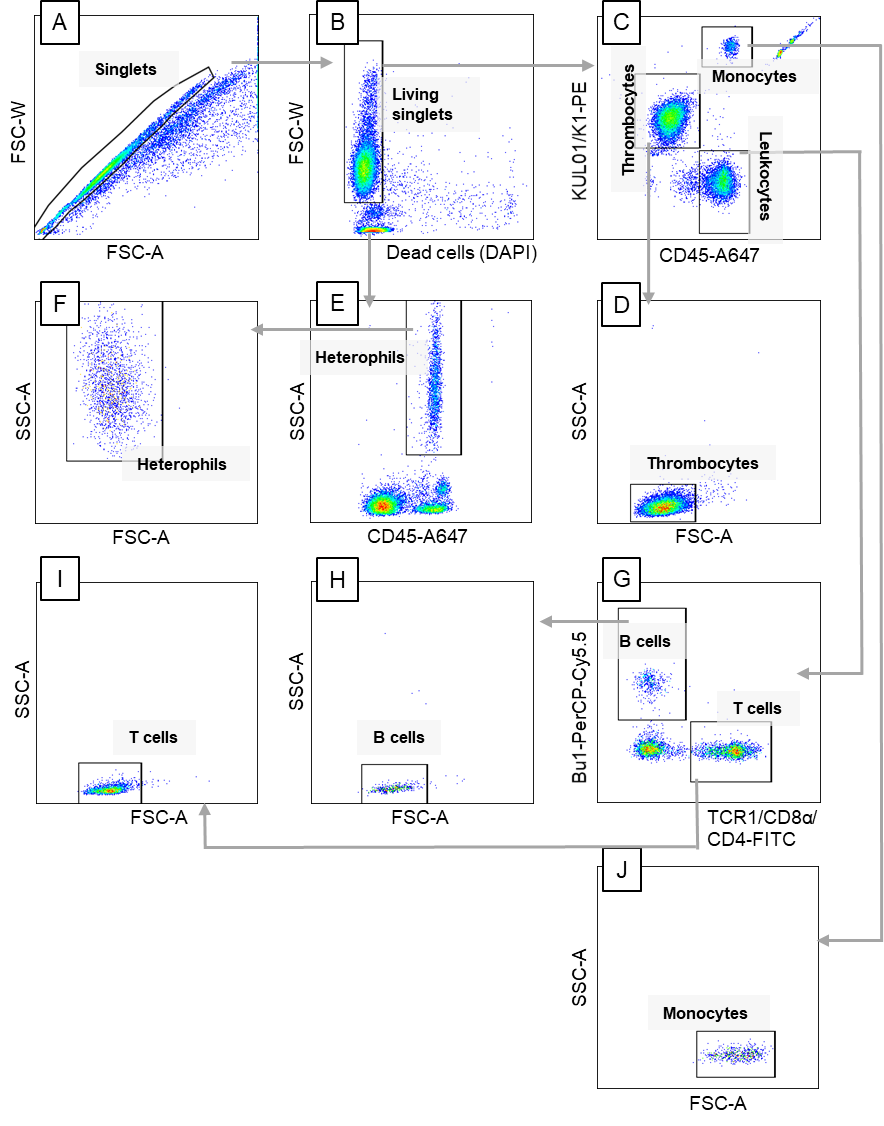


**Supplementary Figure 1. Gating strategy for the discrimination of thrombocytes, monocytes, B cells, T cells and heterophils.** Doublets and dead cells were excluded (A, B) before gating monocytes, thrombocytes, and lymphocytes based on CD45-positive leukocytes, K1-positive thrombocytes, and KUL01-positive monocytes (C). Monocytes and thrombocytes were further validated by back-gating on the SSC/FSC dot plot (D, J). Lymphocytes (B cells and T cells) were identified by the expression of Bu1 or TCR1/CD8α/CD4 (G) and back-gated for verification (H, I). Heterophils were gated on the SSC/CD45 dot plot (E) and back-gated using SSC/FSC (F). Representative data are shown for whole blood from *Salmonella*-infected wild-type chicken at 15 dpi.

**
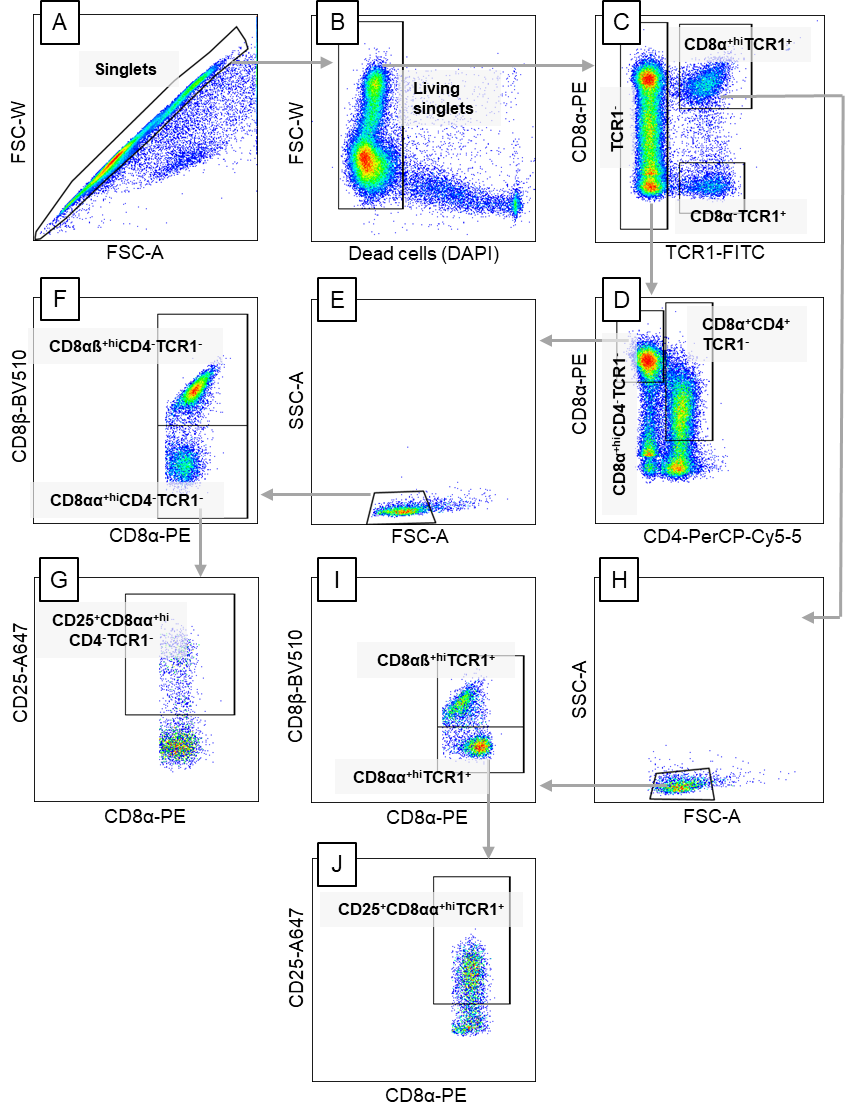
**

**Supplementary Figure 2. Representative gating strategy for identifying TCR1^+^ and TCR1^-^ T cell subsets.** Following the exclusion of doublets and dead cells (A, B), γδ T cells (TCR1^+^) and TCR1^-^ cells were gated and distinguished based on CD8α and TCR1 expression (C). TCR1^-^ cells were further subdivided into CD4-positive and CD4-negative subsets (D). All lymphocyte subpopulations were validated by back-gating using an SSC/FSC dot plot (E, H). CD8α^+hi^TCR1^+^ and CD8α^+hi^CD4^-^TCR1^-^ T cells were further classified into CD8αα-positive and CD8αß-positive subsets (F, I). The activation status of these subsets was assessed by CD25 expression (G, J). Representative data are shown for spleen cells from *Salmonella*-infected wild-type chicken at 12 dpi.


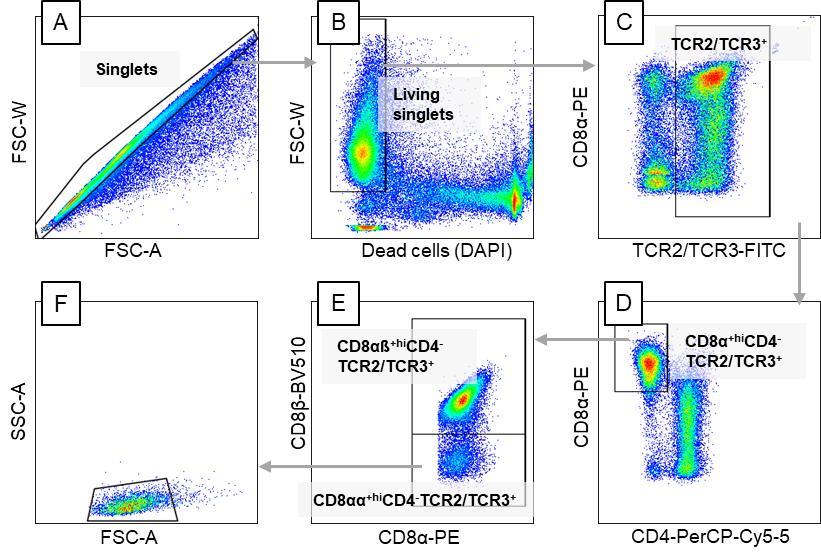


**Supplementary Figure 3. Representative gating strategy for identifying TCR2/TCR3^+^ T cell subsets.** Following the exclusion of doublets and dead cells (A, B), αß T cells (TCR2/TCR3^+^) were gated and distinguished based on CD8α and TCR2/TCR3 expression (C). TCR2/TCR3^+^ cells were further subdivided into CD8α^+hi^CD4^-^TCR2/TCR3^+^ T cells (D), subsequently classified into CD8αα-positive and CD8αß-positive subsets (E). The CD8αα^+hi^CD4^-^TCR2/TCR3^+^ T cell subset was validated by back-gating using an SSC/FSC dot plot (F) Representative data are shown for spleen cells from *Salmonella*-infected knockout chicken at 9 dpi.

**
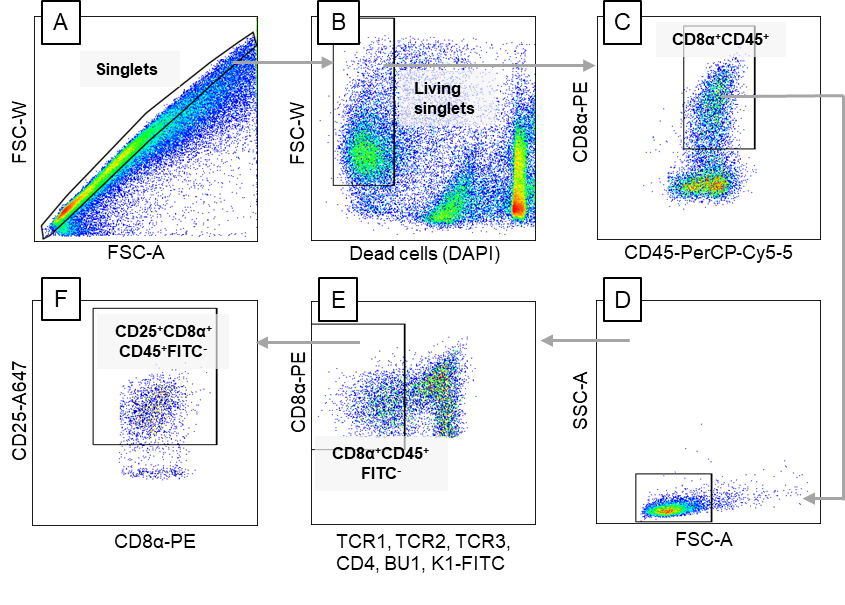
**

**Supplementary Figure 4. Representative gating strategy for identifying NK-like lymphocyte subsets.** Doublets and dead cells were excluded from the analysis (A, B). CD8α^+^ leucocytes (CD45^+^) were gated based on the dot-plot showing CD8α and CD45 expression (C). The lymphocyte subpopulation was confirmed by back-gating using an SSC/FSC dot plot (D). Cells expressing T cell, B cell, and monocyte lineage markers (FITC-conjugated) were excluded, identifying the CD8α^+^CD45^+^FITC^-^ subset (E). The activation status of the CD8α^+^CD45^+^FITC^-^ cell subset was assessed by CD25 expression, visualized on a CD25 versus CD8α plot (F). Representative data are shown for cecum from *Salmonella*-infected knockout chicken at 12 dpi.

**
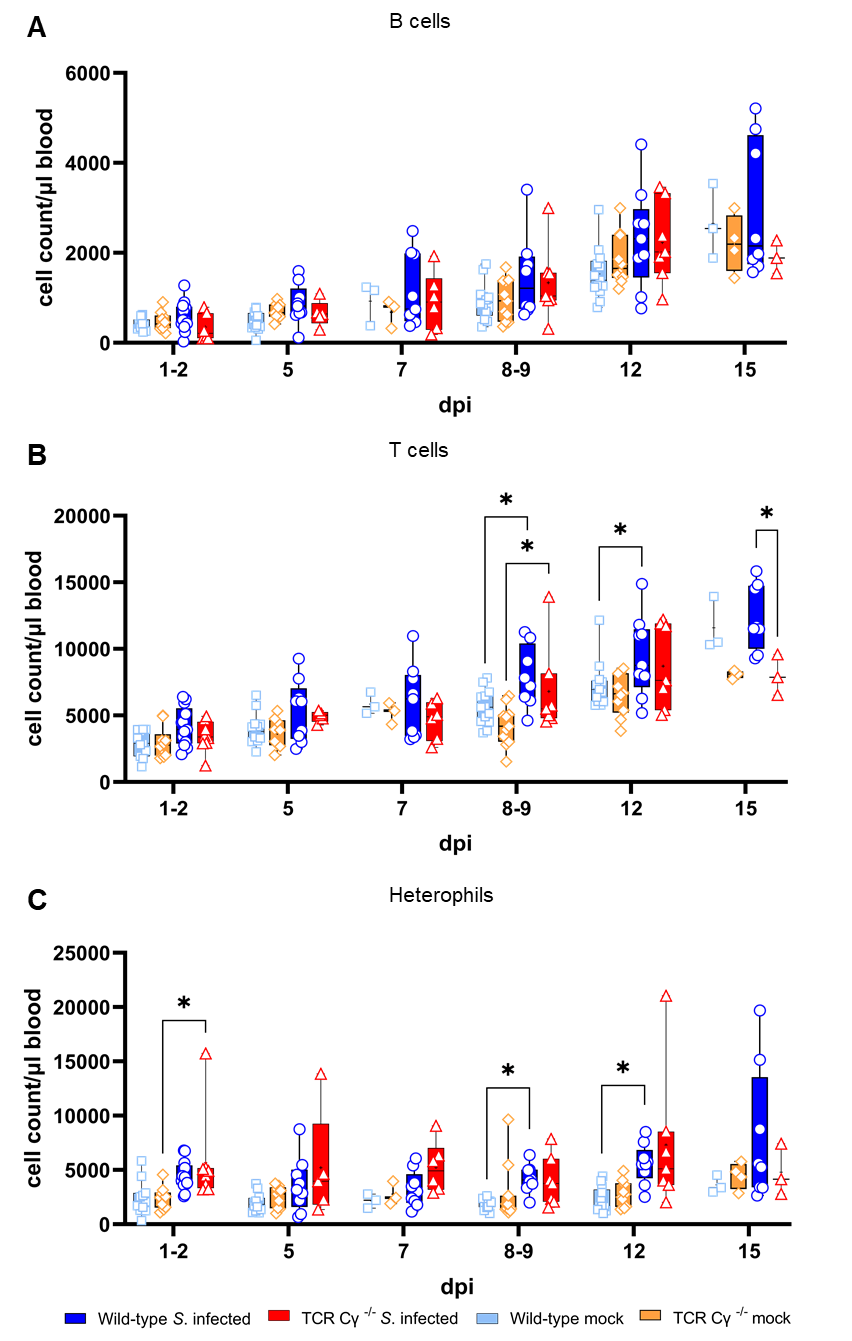
**

**Supplementary Figure 5. Flow cytometric analysis of leukocytes in whole blood following *Salmonella* Enteritidis infection.** Absolute numbers of viable B cells (A), T cells (B) and heterophils (C) were measured over time in blood from wild-type and TCR Cγ^−/−^ chickens following *Salmonella* and mock infection on day 3 of age. Data are presented as minimum and maximum cell counts, with median indicated; n = 2-13. * indicates significant differences between chicken groups, p < 0.05 (B and T cells: 2-way ANOVA-Tukey’s multiple comparison test; heterophils: Mann–Whitney U test).
